# Breath-by-breath comparison of a novel percutaneous phrenic nerve stimulation approach with mechanical ventilation in juvenile pigs: a pilot study

**DOI:** 10.1038/s41598-024-61103-5

**Published:** 2024-05-04

**Authors:** Matthias Manfred Deininger, Dmitrij Ziles, Annegret Borleis, Teresa Seemann, Fabian Erlenkoetter, Christian Bleilevens, Arnhold Lohse, Carl-Friedrich Benner, Steffen Leonhardt, Marian Walter, Thomas Breuer

**Affiliations:** 1 Department of Intensive and Intermediate Care, Medical Faculty, RWTH Aachen University, Pauwelsstr. 30, 52074 Aachen, Germany; 2 Department of Anesthesiology, Medical Faculty, RWTH Aachen University, Aachen, Germany; 3 Chair for Medical Information Technology, Faculty of Electrical Engineering and Information Technology, RWTH Aachen University, Aachen, Germany

**Keywords:** Medical research, Preclinical research, Biomedical engineering, Risk factors, Diseases, Respiratory tract diseases

## Abstract

About one in three critically ill patients requires mechanical ventilation (MV). Prolonged MV, however, results in diaphragmatic weakness, which itself is associated with delayed weaning and increased mortality. Inducing active diaphragmatic contraction via electrical phrenic nerve stimulation (PNS) not only provides the potential to reduce diaphragmatic muscular atrophy but also generates physiological-like ventilation and therefore offers a promising alternative to MV. Reasons why PNS is not yet used in critical care medicine are high procedural invasiveness, insufficient evidence, and lack of side-by-side comparison to MV. This study aims to establish a minimal-invasive percutaneous, bilateral electrode placement approach for sole PNS breathing and thereby enable, for the first time, a breath-by-breath comparison to MV. Six juvenile German Landrace pigs received general anesthesia and orotracheal intubation. Following the novel ultrasound-guided, landmark-based, 4-step approach, two echogenic needles per phrenic nerve were successfully placed. Stimulation effectiveness was evaluated measuring tidal volume, diaphragmatic thickening and tomographic electrical impedance in a breath-by-breath comparison to MV. Following sufficient bilateral phrenic nerve stimulation in all pigs, PNS breaths showed a 2.2-fold increase in diaphragmatic thickening. It induced tidal volumes in the lung-protective range by negative pressure inspiration and improved dorso-caudal regional ventilation in contrast to MV. Our study demonstrated the feasibility of a novel ultrasound-guided, percutaneous phrenic nerve stimulation approach, which generated sufficient tidal volumes and showed more resemblance to physiological breathing than MV in a breath-by-breath comparison.

## Introduction

Critical illness is frequently associated with respiratory dysfunction. Thus, about one-third of all intensive care unit (ICU) patients require sedation, intubation and mechanical ventilation (MV)^1^. About two-thirds of those patients show signs of impaired diaphragmatic contractility and nearly half suffer from delayed ventilator weaning and decreased 2-year survival^2–6^. Levine et al. reported that in brain-dead human organ donors, there was a reduction of about 50% in the diaphragmatic muscular cross-sectional area after less than three days of MV^7^. In rats, on mechanical ventilation, diaphragmatic atrophy could be seen after just 18 hours^8^ and its muscular force could not be recovered via pressure-support ventilation in the weaning process^9^.

This results in a dilemma: Mechanical ventilation is vital for critically ill patients to prevent hypoxemia and treat respiratory failure. However, this is achieved via non-physiologic positive pressure ventilation resulting in diaphragmatic disuse, among other things. Thus, combined lung- and diaphragm-protective ventilation methods have been of growing interest in recent years^10^. Next to modifications in sedation strategy and ventilator settings, phrenic nerve stimulated (PNS) breathing turned out to be a promising future strategy for supplementing or substituting MV^11^.

Up to now, in clinical use PNS has been mainly administered via surgical implantation of phrenic stimulators in patients suffering from central hypopnea syndrome or high spinal cord injuries^12,13^. However, in recent decades, several trans- or percutaneous as well as esophageal methods of electrical or electromagnetic phrenic nerve stimulation have been established and positively evaluated in animals and humans^14–19^. The unifying goal was to establish a minimally invasive, temporary PNS method that provides ventilatory support to critically ill patients^17^. Despite these promising results, to date no direct comparison of the ventilatory characteristics of minimally invasive PNS with MV has been performed.

Therefore, the aim of this study was, firstly, to establish a novel PNS approach using percutaneously placed stimulation electrodes in a porcine model to generate lung-protective tidal volumes and, secondly, to allow the comparison of PNS-induced ventilation in a breath-by-breath comparison with MV.

## Methods

The study was approved by the appropriate governmental institution (Landesamt für Natur, Umwelt und Verbraucherschutz Nordrhein-Westfalen, LANUV NRW, Germany, reference number: 81-02.04.2020.A080) and performed in accordance with German legislation governing animal studies following the “Guide for the care and use of Laboratory Animals” (National Institutes of Health publication, 8th edition, 2011), the principles for care and use of animals based on the Helsinki declaration and the Directive 2010/63/EU on the protection of animals used for scientific purposes (Official Journal of the European Union, 2010). The ARRIVE guidelines (Animal Research: Reporting of In Vivo Experiments) were applied^20^. The experiments were carried out in the Institute for Laboratory Animal Science and Experimental Surgery of the RWTH University Hospital Aachen, Germany.

### Animal model

Six juvenile female German Landrace pigs (*Sus scrofa domesticus*) aged about 3–4 months (body weight [BW] 44 ± 5 kg) were acclimatized in the animal facility for at least two weeks before the experiment. Pigs were used in this experiment, as they seemed to be the most suitable animal model regarding future human transferability. Sex was not expected to have an impact on the outcome of the study endpoints.

### Anesthesia, fluids, and monitoring

The animals were premedicated with intramuscular administration of azaperone (8 mg/kg BW) and atropine (0.2 mg/kg BW). After about 10 min, ketamine (15 mg/kg BW) was injected intramuscularly for sedation. An auricular vein was cannulated, and the induction dosage of pentobarbital (10–20 mg/kg BW) was administered intravenously to achieve an adequate depth of anesthesia for orotracheal intubation (inner diameter 8.5 mm). A total intravenous anesthesia was performed by a continuous intravenous pentobarbital (5–15 mg/kg BW/h) and fentanyl (3–12 µg/kg BW/h) infusion ensuring deep sedation throughout the experiment. An intravenous perioperative antibiotic prophylaxis with 1.5 g cefuroxime (repeated 12-hourly) was administered. To prevent corneal erosion, topical treatment with ophthalmic ointment (dexpanthenol, 5%) was applied to the eyes.

The pigs were positioned supine and an indwelling urinary catheter (Ruesch gold 2-way standard, Teleflex, Athlone, Ireland) was placed. Temperature management was achieved with a convective forced-air warming device (WarmTouch 501–5900, Nellcor, Medtronic, Dublin, Ireland) and two conductive heating pads (EickWarm Heating Pad XL, Eickemeyer, Tuttlingen, Germany). For the entire duration of the experiment, continuous monitoring was performed, including electrocardiogram, oxygen saturation (SpO_2_), temperature and invasive blood pressure measurement (Datex Ohmeda S/5 Anesthesia Monitor, General Electric, Boston, USA). Real-time recording of airway flow and pressure as well as capnometry monitoring (etCO_2_) were performed via ventilator. The depth of anesthesia was clinically monitored by experienced anesthesiologists throughout the experiment. Neuromuscular blocking agents were not performed as this would have disturbed diaphragmatic contraction following phrenic nerve stimulation. Diaphragmatic contraction induced by the pig itself was avoided by deep sedation. Additionally, sonographic assessment was carried out hourly to rule out pig-induced, intrinsic diaphragmatic muscle contractions. Balanced electrolyte solution (4–6 mL/kg BW/h, Sterofundin ISO, B. Braun, Melsungen, Germany) was administered via a central venous catheter in the left external jugular vein. Arterial blood gas analyses (ABL 800, Radiometer, Krefeld, Germany) were performed every two hours.

### Identification of landmarks for ultrasound-guidance of stimulation electrode placement

As phrenic nerve was found to be surrounded by isoechogenic soft tissue and thus too thin to be reliably visualized by transcutaneous ultrasound, one cadaver preparation was performed. This was done as preparation for the in vivo experiments to identify landmarks for ultrasound-guided placement of the stimulation needles in preparation for the in vivo experiments (Supplementary Fig. S1). Therefore, the external jugular vein`s (VJE) junction with the subclavian vein (VS) and on the right side additionally with the left brachiocephalic vein (VBC) were identified as a proper sonographic landmark target area to place the stimulation electrodes near the pigs’ phrenic nerve bilaterally.

### Experimental setup

**Figure 1:**
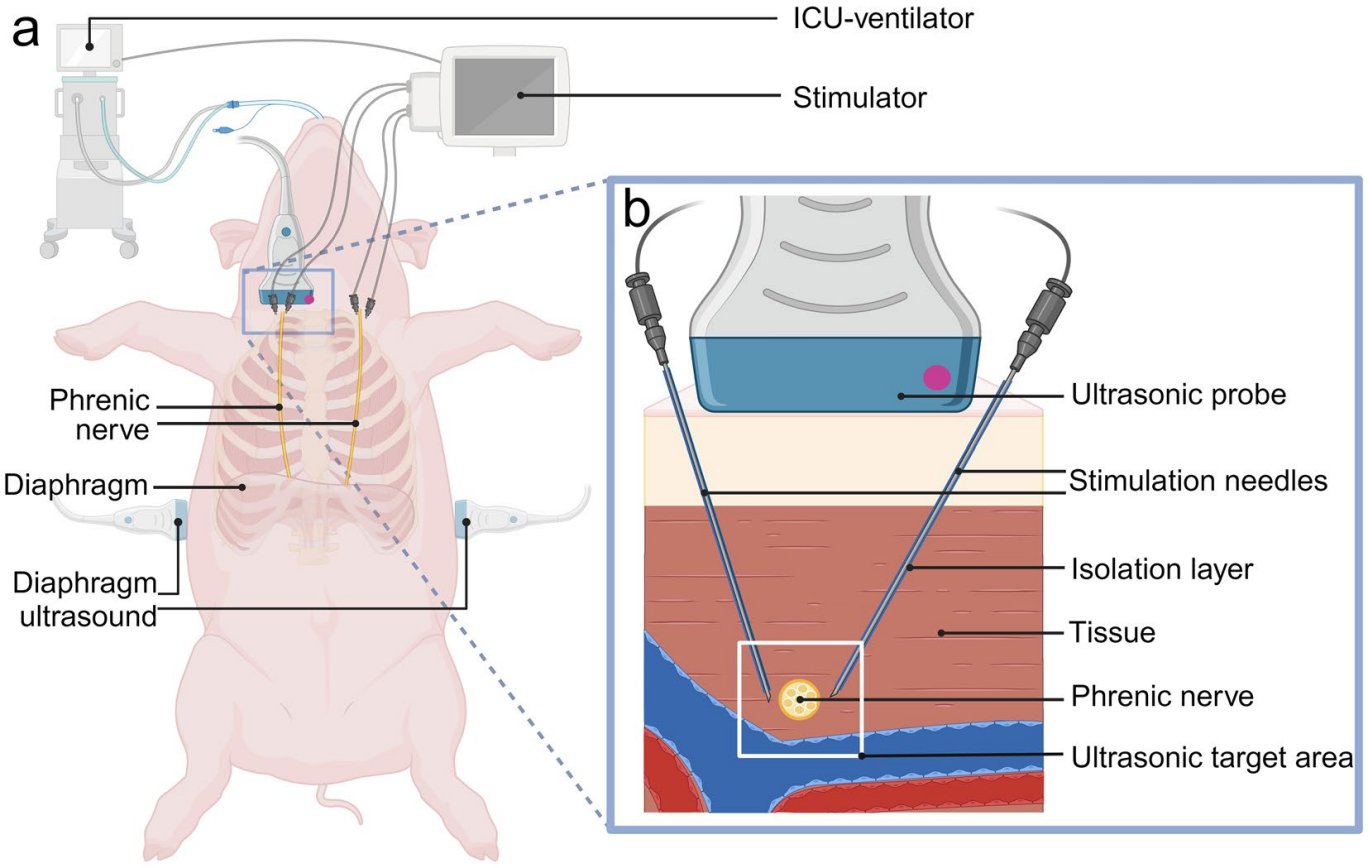
Experimental setup and stimulation electrode placement. (**a**) Shows a schematic illustration of the experimental setup consisting of the intubated pig and an ICU-ventilator connected to the stimulator for phrenic nerve stimulation. The phrenic nerve and the diaphragm are depicted and labeled. The localization of the four stimulation electrodes is outlined in the cervical area of the pig. Additionally, the position for the diaphragm sonography is illustrated on both flank sides. (**b**) Depicts a zoom into the cervical area and shows a cross-section of the stimulation electrodes in correlation to the phrenic nerve. The purple dot indicates the orientation of the ultrasonic probe.

The experimental setup consisted of a custom-built stimulator connected to an ICU-ventilator (EVE IN, Fritz Stephan, Gackenbach, Germany) and percutaneously placed stimulation electrodes^21^. Two stimulation electrodes were inserted per phrenic nerve and, therefore, per side of the body (Fig. 1a). For ultrasound-guidance, a portable ultrasound device with a linear probe (Sonosite Edge II, HFL50x linear probe, 15–6 MHz, FUJIFILM SonoSite Inc., Bothell, USA) was used to locate the sonoanatomical landmarks representing the ultrasonic target area (Fig. 1b).

Echogenic needles which are intended for dual-guided nerve blocks in regional anesthesia (SonoPlex 22 G × 50 mm or 80 mm with facet tip, PAJUNK Medical Produkte GmbH, Geisingen, Germany), were used as stimulation electrodes. These needles allow the administration of liquid from the tip and can be connected to a stimulator directly. To limit the electrical conductivity to the needle tip, the shaft was coated with an electrically insulating layer of acetone and n-butylacetate.

### Ultrasound-guided electrode placement

To enable reliable identification of the ultrasound’s target area and placement of the stimulation electrodes, an ultrasound-guided 4-step approach was developed. Therefore, the ultrasonic linear probe was set in the nerve exam preset with a depth display of about 5–6 cm, as this preset optimizes near-field soft tissue visualization.

**Figure 2:**
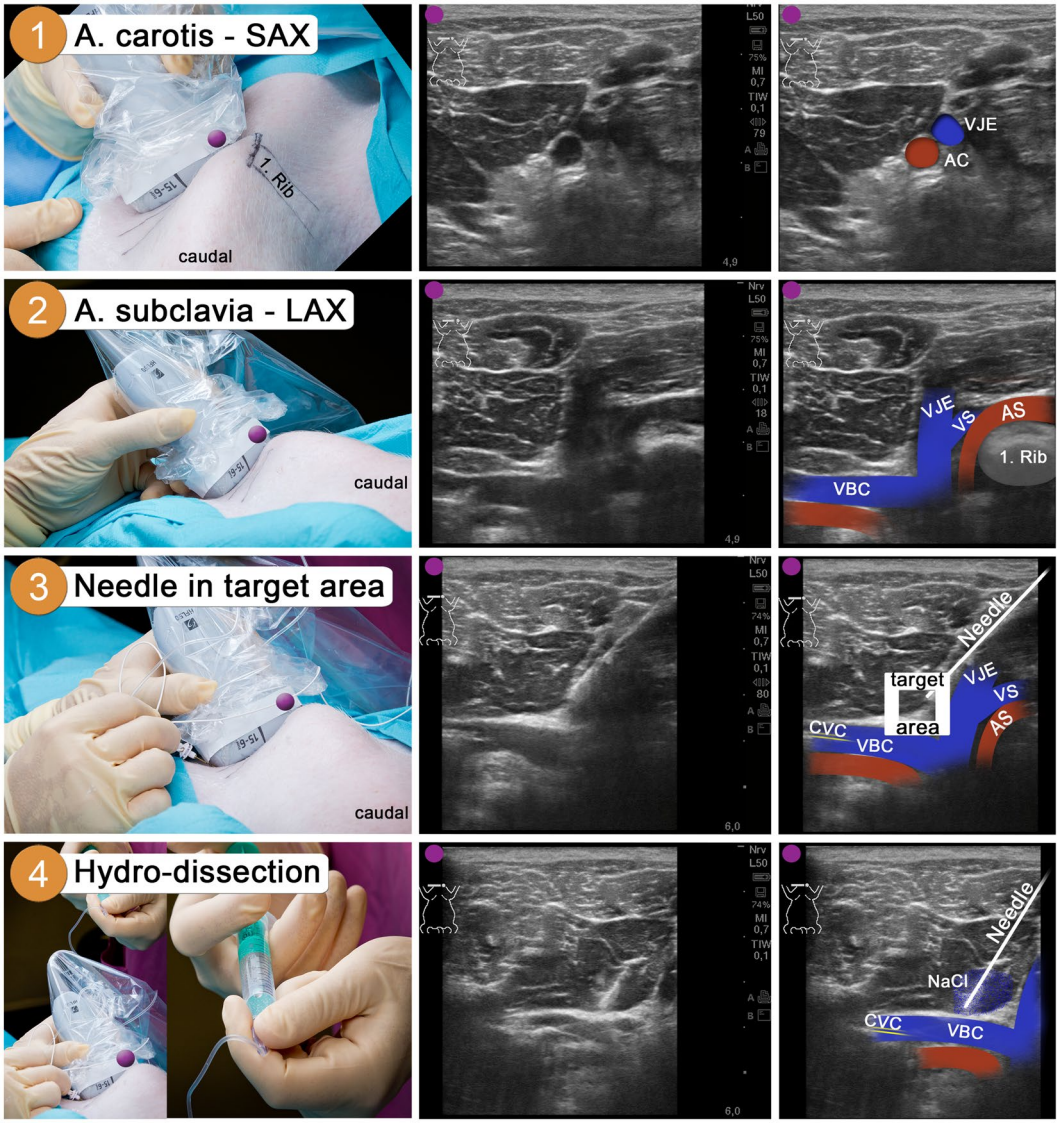
4-step approach for needle placement in the target area on the right side. Left: linear probe position; Middle: corresponding B-mode sonogram; Right: identical sonogram including sonoanatomic labeling. The purple dot indicates orientation of the sonographic probe. Step 1 (picture): Linear probe right above 1. Rib; Step 1 (sonogram): Transversal view on the carotid artery (AC) and the external jugular vein (VJE) in short-axis view (SAX). Step 2 (picture): Tilting probe thoracal to visualize landmarks; Step 2 (sonogram): Oblique view on subclavian artery (AS) and vein (VS) crossing 1. Rib in long-axis view (LAX), confluence of VS with VJE and left brachiocephalic vein (VBC). Step 3 (picture): Needle insertion under aspiration; Step 3 (sonogram): In-plane positioning of the needle in target area (white square). Step 4 (picture): Sodium chloride bolus; Step 4 (sonogram): Hydro-dissection of the phrenic nerve. CVC: Central venous catheter, NaCl: Sodium chloride.

Step 1: The ultrasonic probe was placed on the cranial edge of the first rib to visualize the carotid artery (AC) and the external jugular vein (VJE) in short-axis view (SAX) (Fig. 2, Step 1).

Step 2: The linear probe was tilted caudal until the subclavian artery and vein could be visualized crossing the first rib in long-axis view (LAX). Furthermore, the confluence of VS with VJE was displayed on both sides (Supplementary Video S1). On the right side, additionally, the junction with the contralateral VBC could be seen (Fig. 2, Step 2). The venous confluence restricts the target area (Fig. 2, Step 3 white square; Left side approach: Supplementary Fig. S2).

Step 3: The echogenic needle was inserted under continuous aspiration via in-plane sonographic punction technique (under permanent sonographic visualization) in the target area described earlier (Fig. 2, Step 3).

Step 4: In the last step, a sodium-chloride 0.9% (NaCl) bolus of about 1 ml was administered in the target area for hydro-dissection of the phrenic nerve from the surrounding tissue (Fig. 2, Step 4).

Afterwards, a second echogenic needle was positioned approximately 1 cm medial to the first, again using the ultrasound-guided 4-step approach. The whole procedure was repeated contralaterally to allow bipolar stimulation on both sides. The final optimization of the needle position was performed separately for each phrenic nerve under continuous stimulation to maximize the generated tidal volume. If necessary, the needles were minimally moved (maximal 1–2 mm) in an explorative manner in the puncture channel until the diaphragmatic contraction was maximized.

Complementary to this written explanation an annotated video in the supplements (Supplementary Video S1) illustrates the 4-step approach step-by-step.

### Electrical stimulation method

The bilateral stimulation of the phrenic nerve was performed using a custom-built stimulator designed by the Chair for Medical Information Technology (MedIT, Faculty of Electrical Engineering and Information Technology, RWTH Aachen University, Germany). The stimulation was adjusted depending on diaphragmatic contraction success in the ranges from 5 to 20 V, pulse frequency from 50 to 200 Hz and a pulse length from 50 µs to 4 ms each^21,22^. For a detailed description of the identification of suitable stimulation patterns and settings, we refer to our corresponding publication by Lohse et al.^21^.

### Ventilator settings and stimulation pattern identification

Until monitoring and phrenic nerve stimulation were established, the pigs were ventilated in a lung-protective pressure-controlled MV mode (tidal volume: 4–8 ml/kg BW, Positive end-expiratory pressure (PEEP) 5 cmH_2_O, driving pressure < 15 cmH_2_O; Inspiration:Expiration ratio 1:1 to 1:2; respirator rate: 20–26, end-tidal CO_2_ 35–45 mmHg, Fraction of inspired oxygen (FiO_2_): 0.3) by the ICU-ventilator. The stimulation electrode placement was followed by iterative testing of different PNS stimulation patterns to identify the most effective ones as described in our previous paper. In brief, the stimulation pattern identification was primarily based on the achievable tidal volume. Secondly, when evaluating stimulation patterns with the same tidal volume, a lower pulse length and pulse frequency was preferred, as this results in less electrical energy output to surrounding tissue and the phrenic nerve. In addition, a longer voltage rise time was positively rated, as it was associated with a smoother inspiratory muscle contraction^21^.

The first pig (Pig1) was primarily used for the initial establishment of the ultrasound-guided PNS breathing and systematic stimulation pattern testing. From the second pig (Pig2) onwards, six hours of the 24 h experimental period were used for the breath-by-breath comparison with MV.

During PNS, the ICU-ventilator was set to continuous positive airway pressure (CPAP) mode with a PEEP of 5 cmH_2_O to avoid atelectasis. However, no pressure support was administered by the ICU-ventilator to allow quantification of PNS-induced diaphragmatic contraction via resulting tidal volumes. After completion of the exploratory stimulation pattern identification, a breath-by-breath alternation between MV and PNS was performed to allow direct comparison of the two ventilation modes. The target tidal volume for phrenic nerve stimulated breathing was set and modulated between 4 and 8 ml/kg BW, to meet the criteria for lung-protective ventilation^23^. This tidal volume is primarily administered in patients suffering from acute respiratory distress syndrome (ARDS)^24^.

#### Quantification of diaphragmatic muscular contraction

The sonographic Brightness- (B-mode) and Motion-mode (M-mode) were used to visualize and evaluate the sufficiency of the diaphragmatic contraction by phrenic nerve stimulation. For quantification of diaphragmatic thickening, the linear ultrasound probe was placed with craniocaudal orientation on the anterior-axillary line in the zone of diaphragmatic apposition to the thoracic wall and M-mode was recorded (position of the sonographic probe is illustrated in Fig. 1a)^25^. Subsequently, the calculation of the ratio between end-inspiratory and end-expiratory diaphragm thickness (d_I_/d_E_-ratio) was performed bilaterally from the third pig onwards at two different time points (at the beginning of the experiment and after about twelve hours). To reduce observer bias, quantification was carried out by two authors (MMD, DZ), blind to each other.

#### Electrical impedance tomography

Electrical impedance tomography (EIT, PulmoVista 500, Draeger, Luebeck, Germany) was used in two pigs (Pig5, Pig6) to study the regional air distribution in the lungs during ventilation. For this purpose, the EIT belt was placed, so that caudal parts of the lungs were visualized, as dorso-caudal regional ventilation was expected to be reduced, at least during mechanical ventilation^26^. For the qualitative analysis of EIT impedance signals, data was analyzed using the manufacturer’s software (Draeger EIT Data Analysis Tool 6.1, Draeger, Luebeck, Germany). To quantify the regional distribution of tidal volumes in the caudal lung areas, the geometrical Center of Ventilation (CoV) in EIT was calculated using a custom-built MATLAB script. The CoV is described by x- and y-coordinate pairs each between 0 and 1. For this study, the y-value was relevant. It describes the ventro-dorsal orientation, where larger values represent a more dorsally located CoV^27^. The CoV was calculated for 300 breaths of PNS as well as MV separately in a 1:1 breath-by-breath comparison.

#### Pressure–volume curves

To describe the relationship between pulmonary ventilation pressure and tidal volume during inspiration and expiration, corresponding curves were calculated. Therefore, from the second pig (Pig2) onwards for each animal automatically recorded ventilator data for 100 stimulated and 100 mechanical breaths were analyzed, and the mean pressure–volume curves were visualized using a custom-built MATLAB script. To determine the effective ventilation pressure applied to the lungs, also called intratracheal pressure (Ptrach), the corresponding airway pressure (Paw) was corrected for artificial endotracheal tube and breathing filter-related resistances as first described by Guttmann et al^28,29^. Mean intratracheal pressure values for in- and expiration were calculated for 25%, 50% and 75% of the average PNS-induced tidal volume for statistical analysis.

### Euthanasia

Animals were under deep anesthesia as well as analgesia for the entire experiment. They were euthanized without regaining consciousness by an intravenous application of a lethal dose of pentobarbital (160 mg/kg BW) and potassium chloride (75 mg/kg BW) in accordance with the study protocol.

### Data collection, analysis, and statistics

Data collection was performed using commercially available hard- and software (hardware: MicroLabBox, dSPACE GmbH, Paderborn, Germany; software: ControlDesk 7.1, dSPACE GmbH, Paderborn, Germany; MATLAB Version R2022b, MathWorks, Natick, USA). All data from the ICU-ventilator was collected using custom-built software developed at the Chair for Medical Information Technology (MedIT, Faculty of Electrical Engineering and Information Technology, RWTH Aachen University, Germany) as published in 2020^30^.

Data analysis was performed using commercially available software packages (Microsoft Excel, Microsoft 365 MSO, Version 2112, Redmond, USA; MATLAB Version R2022b, MathWorks, Natick, USA). From the second animal onwards, tidal volumes were quantified by area-under-curve calculation using flow-curve data in MATLAB and aggregated as mean per hour.

Statistics were performed using GraphPad Prism 10 (Version 10.0.3, GraphPad Software Inc., San Diego, USA) and SPSS version 28 (IBM Corp., Armonk, NY). Paired t-test was used for parametric values. Simple linear regression was performed to calculate the slope of tidal volume change over experimental time. When appropriate, data is presented as mean ± SD, median (range), absolute numbers or for the slope, as value with 95% confidence interval [95%-CI]. All statistical tests are two-tailed. Significance was defined as p < 0.05. Since this was an exploratory study and thus the effect size was unknown, no a priori power analysis could be performed. However, based on previous investigations of phrenic nerve stimulation in pigs^31,32^, it was expected that feasibility could be shown reliably with a respective group size of n = 6 animals.

**Figure 3:**
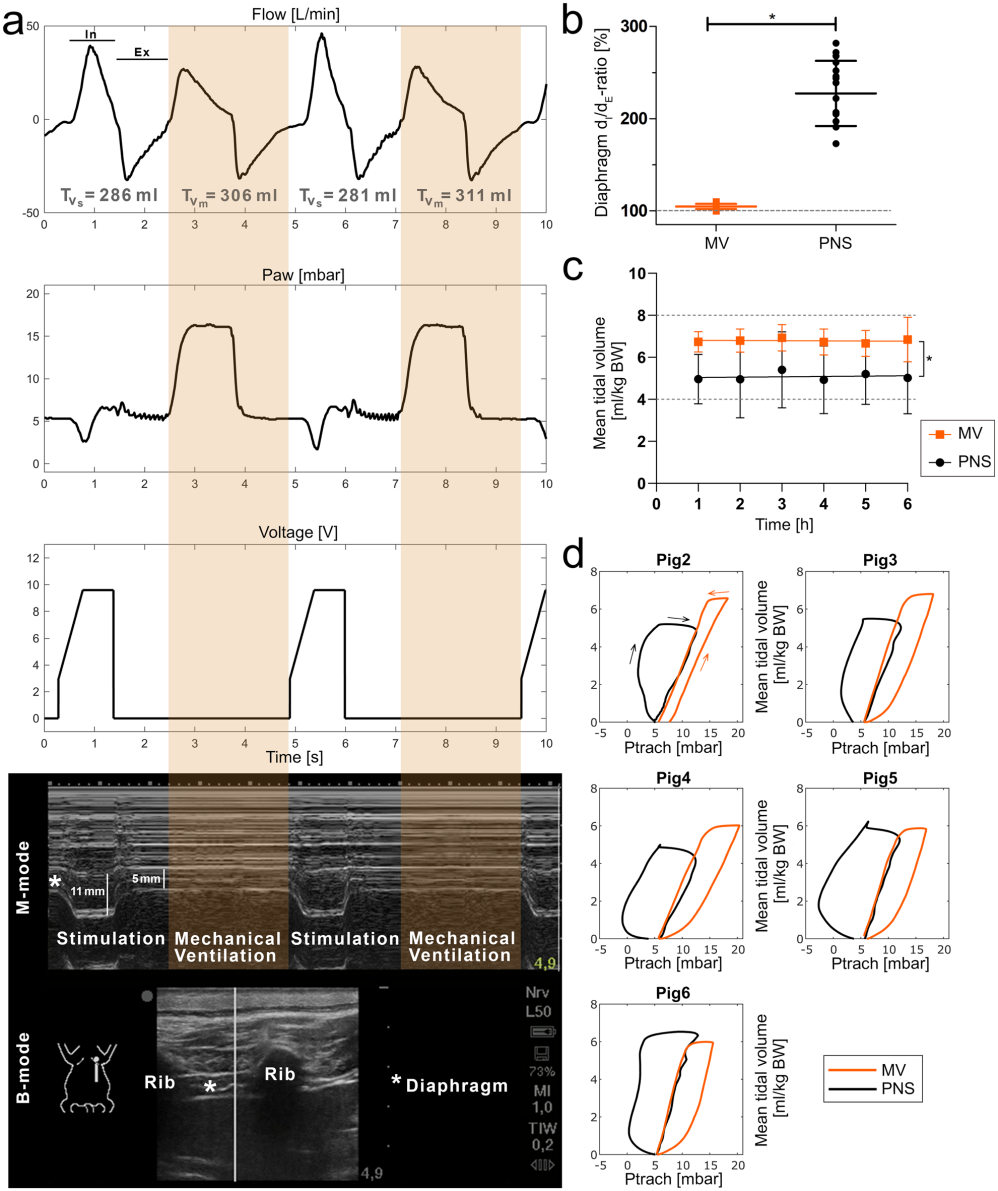
Comparison of mechanical ventilated and phrenic nerve stimulated breaths. (**a**) Flow, airway pressure, stimulation voltage and left-side diaphragmatic sonography are plotted over a 10 s period (Pig6, BW: 49 kg; target tidal volume set: 294 ml [6 ml/kg BW]). Phrenic nerve stimulated breaths (PNS, white background) were solely induced by bilateral electric stimulation of the phrenic nerves (here 9.5 V, see voltage curve). This resulted in the contraction of the diaphragm with a 2.2-fold inspiratory-thickening [M-mode diaphragm: 11 mm (end-inspiratory diameter, d_I_); 5 mm (end-expiratory diameter, d_E_)], which led to a decrease in airway pressure (Paw) and consecutive inspiratory flow. Mechanical ventilated breaths (MV; orange background) were applied using pressure-controlled ventilator mode. The exact tidal volume is indicated for each PNS (Tv_s_) and MV breath (Tv_m_). (**b**) The ratio between end-inspiratory and end-expiratory diaphragm thickness (d_I_/d_E_-ratio) was quantified bilaterally at two time points by two investigators independently. The baseline (dashed grey line) represents the respective end-expiratory diaphragmatic thickness (100%). Data from each measurement is plotted individually, mean ± SD is indicated (n = 4^+^). (**c**) Over six hours, the mean values for all MV and PNS breaths per hour (normalized to the body weight of the respective pig) were plotted separately (n = 5). The horizontal continuous lines illustrate the respective trend over time. The dashed grey lines indicate the target corridor for lung-protective ventilation (4-8 ml/kg BW). (**d**) The averaged intratracheal pressure (Ptrach)-volume curves were calculated for PNS and MV (100 consecutive breaths per ventilation mode) separately for each pig (n = 5). ^+^systematic sonographic quantification not performed for the second pig (Pig2), *p < 0.001. *Ex* expiration, *In* inspiration, *Paw* airway pressure, *Ptrach* intratracheal pressure, *Tv*_*s*_ phrenic nerve stimulated tidal volume, *Tv*_*m*_ mechanical ventilated tidal volume.

**Figure 4:**
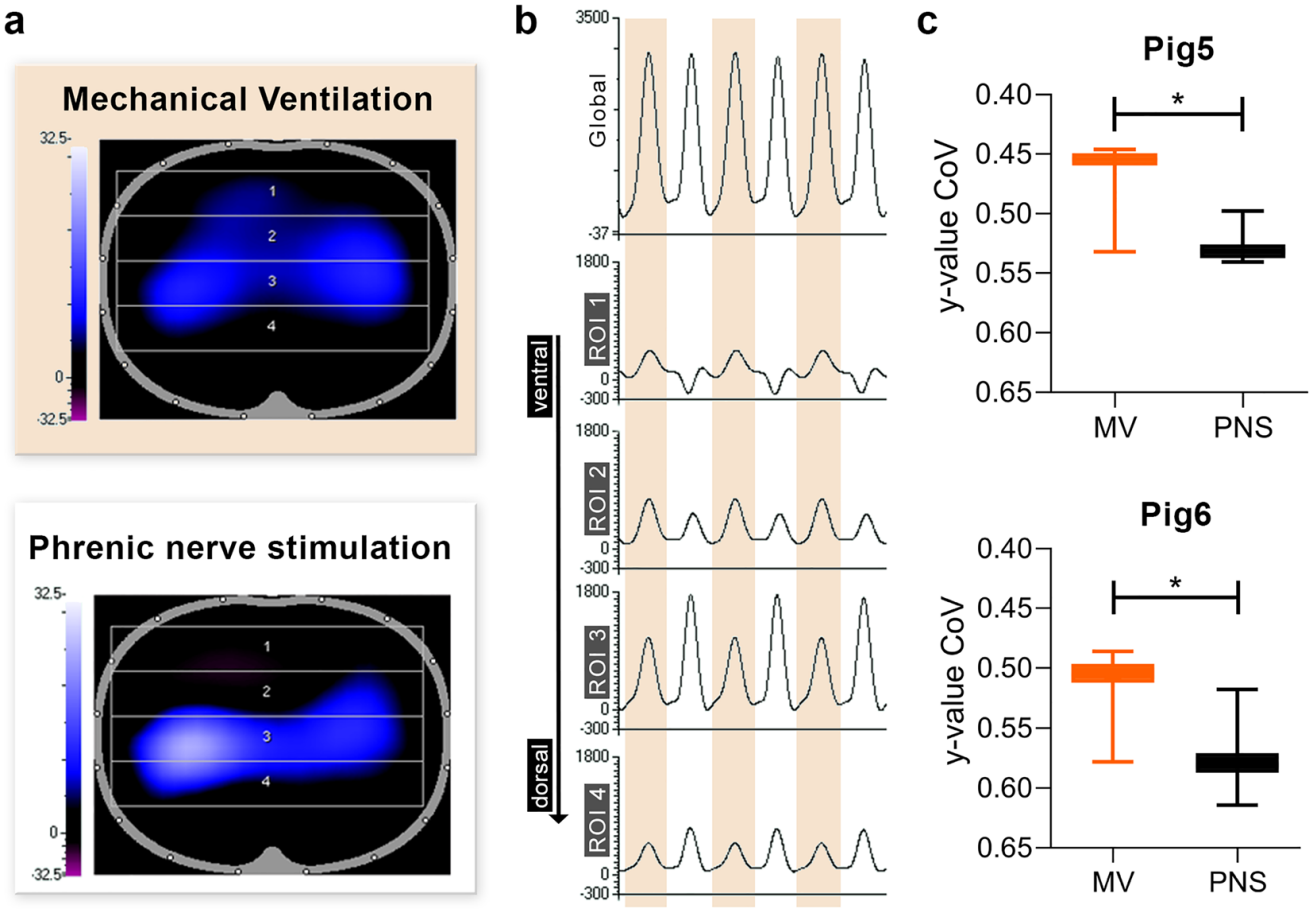
EIT-based evaluation of caudal ventilation in PNS and MV. (**a**) Shows a qualitative representation of inspiration for MV (top, orange background) and PNS (bottom, white background) in relation to the chest cross-section. The stronger the intensity of the EIT signal, the brighter the blue color. Regions of interest (ROI) analyzed in (**b**) are shown as numbered rectangles, where region 1 is ventral and region 4 is caudal. (**b**) Depicts an exemplary EIT quantification for six breaths. The EIT impedance intensity (y-axis) is displayed over time (x-axis). In addition, to the global EIT-signal depicted in the top graph, a differentiation and quantification of the regional EIT-signal in four layers (ROIs) from ventral to dorsal was performed. A breath-by-breath comparison illustrates differences in regional EIT-signal between MV (orange background) and PNS (white background) breaths. (**c**) Shows the comparison of the Center of Ventilation (CoV) for MV and PNS, where higher y-values indicate a more dorsal location of the CoV. The CoV was calculated separately for 300 PNS and 300 MV breaths, which alternated breath-by-breath. Ventral is at the top in all figures/ROIs. *p < 0.001. *CoV* center of ventilation, *EIT* electrical impedance tomography, *MV* mechanical ventilation, *PNS* phrenic nerve stimulation, *ROI* Region of interest.

Figure 1 was created using BioRender.com. Figures 2, 3, 4 and Supplementary Figs. S1 and S2 were compiled and annotated using Photoshop CS6 (Version: 13.0). Shotcut Video Editor (Shotcut, Version 23.11.29, Meltytech, Oceanside, USA) was used to compile the supplementary videos. Sonograms and B-mode video-loops were captured using the portable ultrasound device with a linear probe (Sonosite Edge II, HFL50x linear probe, 15–6 MHz, FUJIFILM SonoSite Inc., Bothell, USA).

## Results

### Feasibility of ultrasound-guided phrenic nerve stimulation

In all six juvenile pigs, invasive monitoring and bilateral stimulation of the phrenic nerve and subsequent bilateral diaphragmatic contraction could be established in median within two hours in total (range 1.5–3.5 h) using ultrasound-guided needle placement (Fig. 1).

No experiment had to be terminated due to an adverse event. However, the first animal (Pig1) suffered from hemodynamically relevant septic or anaphylactic shock of unknown origin immediately after induction of anesthesia. After fluid, catecholamine and antibiotic administration, as well as electric cardioversion, the animal recovered. This allowed the experiment to proceed after a four-hour delay in accordance with local animal welfare authorities. The third pig (Pig3) developed a pneumothorax most likely due to attempts to optimize stimulation electrode placement. This required the placement of a chest tube on the affected side. After this procedure, the experiment could be completed without further complications.

In all pigs hemodynamic and respiratory values were stable throughout the entire experiment (Supplementary Fig. S3). Peripheral oxygen saturation measured at the pig’s tail was above 95% over the entire experimental period. Also, the two-hourly arterial blood gas analysis showed stable, physiological values under mean FiO_2_ of 0.31 (± 0.04) (Supplementary Table S1).

### Sufficient diaphragmatic contraction and ventilation via phrenic nerve stimulated breaths

Based on the exploratory approach of the project, the first pig (Pig1) was primarily used to establish the PNS-induced ventilation. Consequently, systematic quantitative, comparative analyses between PNS and MV were performed from the second experimental animal onwards. There was no evidence of nervous co-stimulation, especially of the nearby vagus nerve, nor muscular side effects, in any of the six pigs. To compare the effects of PNS and MV on diaphragm contractility and ventilation, a breath-by-breath alternation was performed.

Diaphragmatic contraction showed a significant end-inspiratory to end-expiratory thickening (d_I_/d_E_-ratio) for PNS when compared to MV (PNS d_I_/d_E_-ratio: 224 ± 34%; MV d_I_/d_E_-ratio: 104 ± 3%, p < 0.001; Fig. 3a,b; Supplementary Video S2). The tidal volume resulting from phrenic nerve stimulation was in the lung-protective range, but significantly lower than the tidal volume generated via MV (PNS: 5.1 ± 1.6 ml/kg BW, MV: 6.8 ± 0.6 ml/kg BW, p < 0.001; Fig. 3a,c). Over the observation period of six hours, there was no significant change in PNS nor MV mean hourly tidal volume according to the slope in simple regression analysis (PNS: 0.016 [95% CI: -0.314 to 0.346, p = 0.92]; MV: -0.008 [95% CI: -0.165 to 0.149, p = 0.92]). The exemplary airway pressure over time curve (Fig. 3a) as well as the intratracheal pressure–volume curves (Fig. 3d) illustrate from a qualitative perspective the negative inspiratory pressure for PNS compared to the positive pressure for MV breaths. The quantitative analysis of the intratracheal pressures at 25%, 50% and 75% of the tidal volume showed significant differences between PNS and MV for both the in- and expiration phases (Supplementary Table S2).

### Improved ventilation of dorso-caudal lung areas for phrenic nerve stimulated breaths

To further characterize the relevance of active diaphragmatic contraction in PNS for caudal regional air distribution, we analyzed MV and PNS breaths separately using EIT for the last two animals (Pig5 and Pig6).

The exemplary depicted qualitative analysis in Fig. 4a (Supplementary Video S3) shows a discrepant regional distribution of the EIT signal for MV and PNS. To better describe this effect, four regions of interest (ROI) from ventral to dorsal were defined. The change in impedance per ROI and breath was analyzed separately (Fig. 4b). Despite globally comparable impedances between PNS and MV, in direct comparison of ROIs, PNS breaths showed lower impedance changes in the ventrally located ROIs and higher in the dorsally located ROIs when compared to MV breaths. For quantification, the y-value of the CoV indicating ventro-dorsal distribution was calculated and showed significantly more dorsal ventilation for PNS compared to MV (PNS: 0.55 ± 0.03; MV: 0.48 ± 0.03, p < 0.001, Fig. 4c shows the separate values for Pig5 and Pig6).

## Discussion

In recent years, an increasing number of studies have been published that augment MV with minimally- or non-invasive PNS^15–17,19,33,34^. The results of these studies are promising as they showed beneficial effects of PNS regarding diaphragmatic atrophy, decreasing atelectasis and MV pressures in pig model and partially in human patients^18,32,34–37^. Thus, PNS appears promising as a diaphragmatic- and lung-protective ventilation method^35^.

To our knowledge, this study is the first to compare the aspects of pure minimally invasive PNS with MV. So far, PNS was primarily used to augment MV, achieving an increase of up to one-third (35%) in tidal volume or inspiration pressure^33,34,38^. Therefore, the effects of PNS on diaphragmatic contractility, pressures or regional ventilation could consistently be evaluated only to a limited extent.

In our study sole PNS resulted in negative pressure inspiration, due to its active diaphragm contraction, leading to significantly improved ventilation of dorso-caudal lung areas compared to MV. The results suggest that PNS imitates spontaneous breathing more closely than MV. However, it does not activate additional breathing muscles that decrease the diaphragmatic work of breathing during physiological spontaneous breathing^39^. Recently, two feasibility studies by Mueller et al. and Panelli et al. showed, analog to our data, for electromagnetic-induced PNS alone that lung-protective tidal volumes can be generated by negative pressure inspiration. Although this was demonstrated in both studies for periods of only 10 breaths each in anesthetized humans^16,19^. Concordant with our findings, studies in healthy pigs and human ARDS patients showed a tendency in EIT to shift regional lung ventilation from ventral to dorsal and reduce airway pressures using intermittent transvenous PNS augmentation of MV^33,35^.

The percutaneous, ultrasound-guided stimulation approach established in this study appears to add value compared to existing PNS methods as it could be established in all six pigs bilaterally and allowed stable stimulated breathing in alternation with MV for at least 6 h. Previous studies report an error rate (one-sided or no stimulation) up to 13%^18^ and variability in phrenic nerve stimulation strength^32^. Percutaneous approaches described so far applied the stimulating electrodes from dorsal through neck access in a wire-over-needle technique. In terms of transition to ICU patients, as these are mostly in supine position, ventral access as described in this study might offer better accessibility and shorter needle insertion depth, whereas the atraumatic stimulation electrode inserted by O’Rourke et al. appears to be advantageous due to the lower potential for complications.

Hence, we note that our study has several limitations. Firstly, the stimulation period of this feasibility study was too short to determine positive or harmful effects of phrenic nerve stimulated breathing on the lungs or diaphragm, possible phrenic nerve damage from the electrical stimulation as well as other relevant outcome parameters. Secondly, the stimulation pattern used, led to a relatively short, strong diaphragmatic contraction, which consequently mimics physiological spontaneous breathing only to a limited extent. The stimulation pattern should therefore be optimized in further studies. Thirdly, a breath-by-breath switch between MV and PNS was used, to directly compare airway pressure, flow, and generated tidal volume. Consequently, the current study does not allow a statement about sufficiency of stimulated ventilation alone for longer periods. To address these aspects, long-term phrenic nerve stimulated breathing, in comparison to a mechanically ventilated control group, would be valuable. Fourthly, the phrenic nerve itself could not be visualized with our study`s transcutaneous ultrasound, which led to a landmark-based needle placement approach with subsequent optimization under stimulation. Nonetheless, in all six pigs, sufficient bilateral stimulated breathing was possible. However, fifthly, in one pig, this needle optimization led to a unilateral pneumothorax, which had to be relieved by inserting a chest tube. Every invasive procedure, where a needle is placed close to the thorax, is associated with this risk. For long-term usage, the insertion of a non-traumatic electrode for stimulation might be favorable for optimal risk reduction. Sixthly, the study was performed in a small number of preclinical healthy pig model as a proof-of-concept and does not imply transferability to disease models or procedural feasibility in humans at this time. For this purpose—after the safety and functionality has been tested in animal models extensively—a first-in-human trial should be aimed for in the future.

## Conclusions

We successfully implemented a novel ultrasound-guided percutaneous approach for bilateral phrenic nerve stimulation generating sufficient tidal volumes. The subsequent breath-by-breath comparison with MV revealed a more physiological-like ventilation for PNS breaths. To determine whether it could be a valuable ventilation method for ICU patients in the future, further investigations might be valuable to evaluate the feasibility of PNS breathing over extended periods.

### Supplementary Information

Supplementary Information 1.

Supplementary Video 1.

Supplementary Video 2.

Supplementary Video 3.

## Data Availability

The datasets used and/or analyzed during the current study are available from the corresponding author on reasonable request.
