# Supplementary material for: Breath-by-breath comparison of a novel percutaneous phrenic nerve stimulation approach with mechanical ventilation in juvenile pigs: a pilot study
Source: Sci Rep. 2024 May 4;14:10252. doi: 10.1038/s41598-024-61103-5 (PMC11069575; doi:10.1038/s41598-024-61103-5)
Supplement: Supplementary file 1 — Supplementary Information 1. [file 41598_2024_61103_MOESM1_ESM.pdf]

Supplementary material

Breath-by-breath comparison of a novel percutaneous phrenic nerve stimulation approach with mechanical ventilation in juvenile pigs: a pilot study

Matthias Manfred Deininger[1*]; Dmitrij Ziles[1]; Annegret Borleis[1]; Teresa Seemann[1]; Fabian Erlenkoetter[1]; Christian Bleilevens[2]; Arnhold Lohse[3]; Carl-Friedrich Benner[3]; Steffen Leonhardt[3]; Marian Walter[3]; Thomas Breuer[1]

Affiliations:

[1] Department of Intensive and Intermediate Care, Medical Faculty, RWTH Aachen University, Aachen, Germany

[2] Department of Anesthesiology, Medical Faculty, RWTH Aachen University, Aachen, Germany

[3] Chair for Medical Information Technology, Helmholtz-Institute for Biomedical Engineering, RWTH Aachen University, Aachen, Germany

[*] corresponding author: mdeininger@ukaachen.de

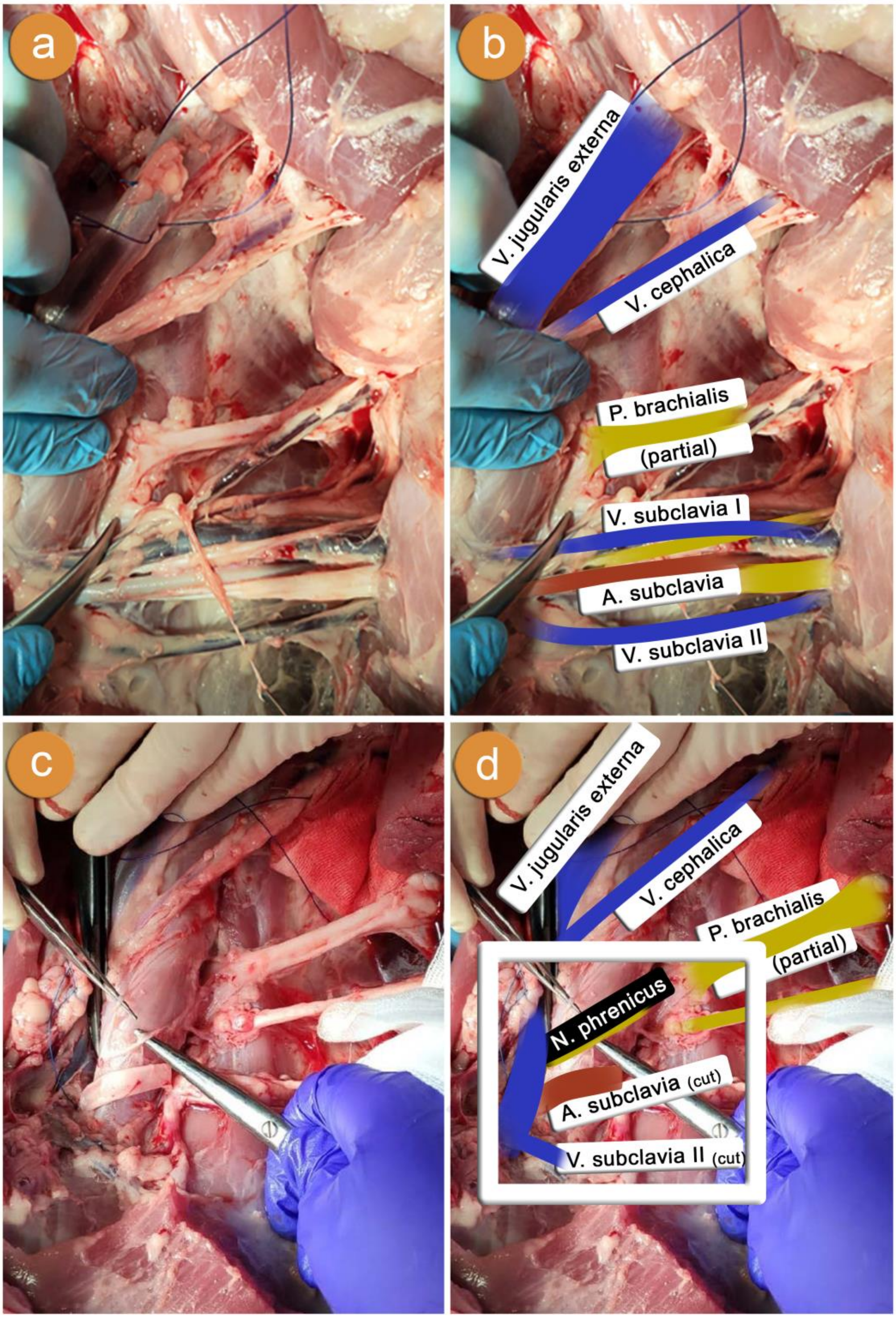

Fig. S1: Lateral view on porcine left collar region in supine position after preparation

The top of each picture is cranial. a) and c) show the region of interest excluding, b) and d) including relevant annotations. Sections a) and b) show inter alia the brachial vessel nerve bundle (Plexus brachialis) that has been separated in sections c) and d) to expose the phrenic nerve (Nervus phrenicus) and the landmark vessels (Vena jugularis externa, Vena/Arteria subclavia) used for identification of the target area (white square) for sonographic needle-placement.

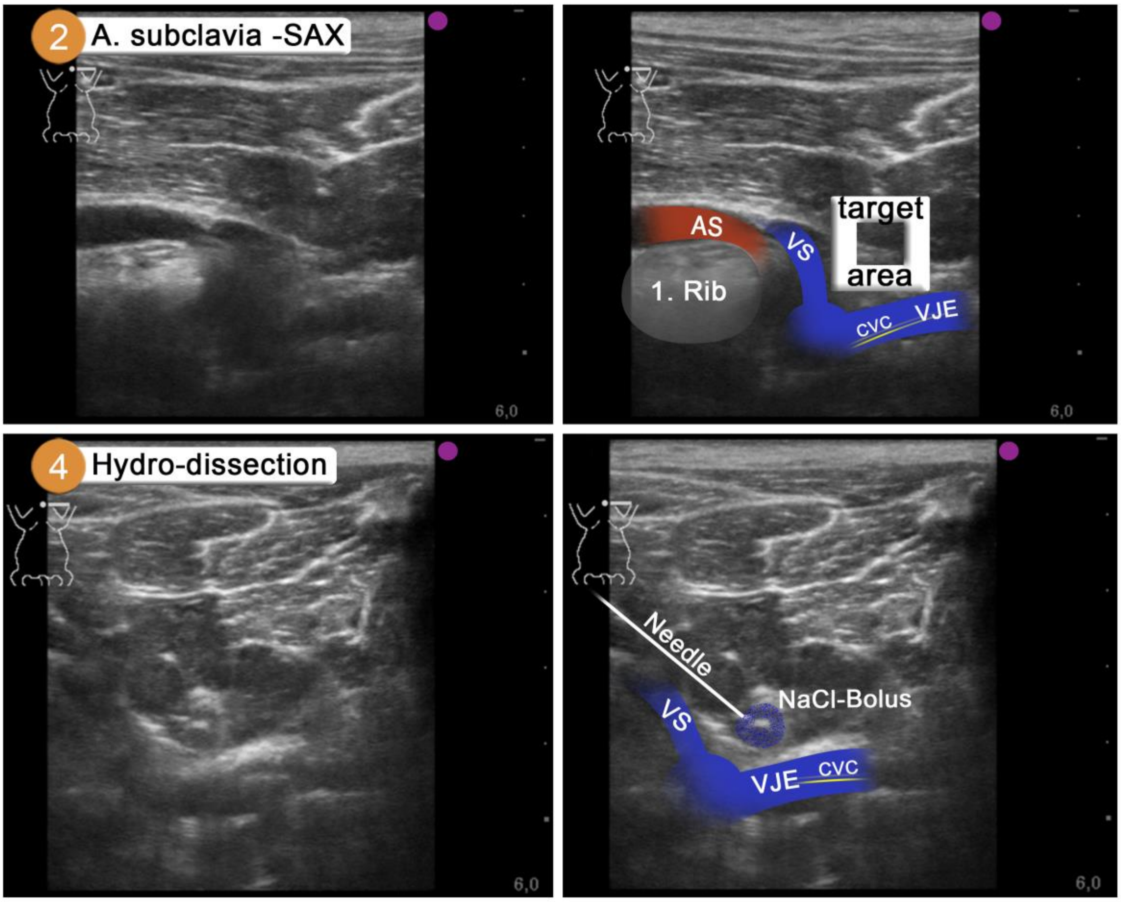

Fig. S2: Ultrasound-guided 4-step approach for needle placement in the target area on the left side

Left: B-mode sonogram; Right: Identical B-mode sonogram including sonoanatomic labeling. Purple dot indicates sonographic orientation. Steps 1 and 3 are identical to the right side (see main manuscript, Fig. 2); Step 2: Oblique view on subclavian artery (AS) and vein (VS) crossing 1. rib in long-axis view (LAX), confluence of VS with the external jugular vein (VJE) restricting the target area (white square); Step 4: Hydro-dissection of phrenic nerve after needle insertion in the target area.
AS: Subclavian artery, CVC: Central venous catheter, NaCl: Sodium chloride, VJE: External jugular vein, VS: Subclavian vein

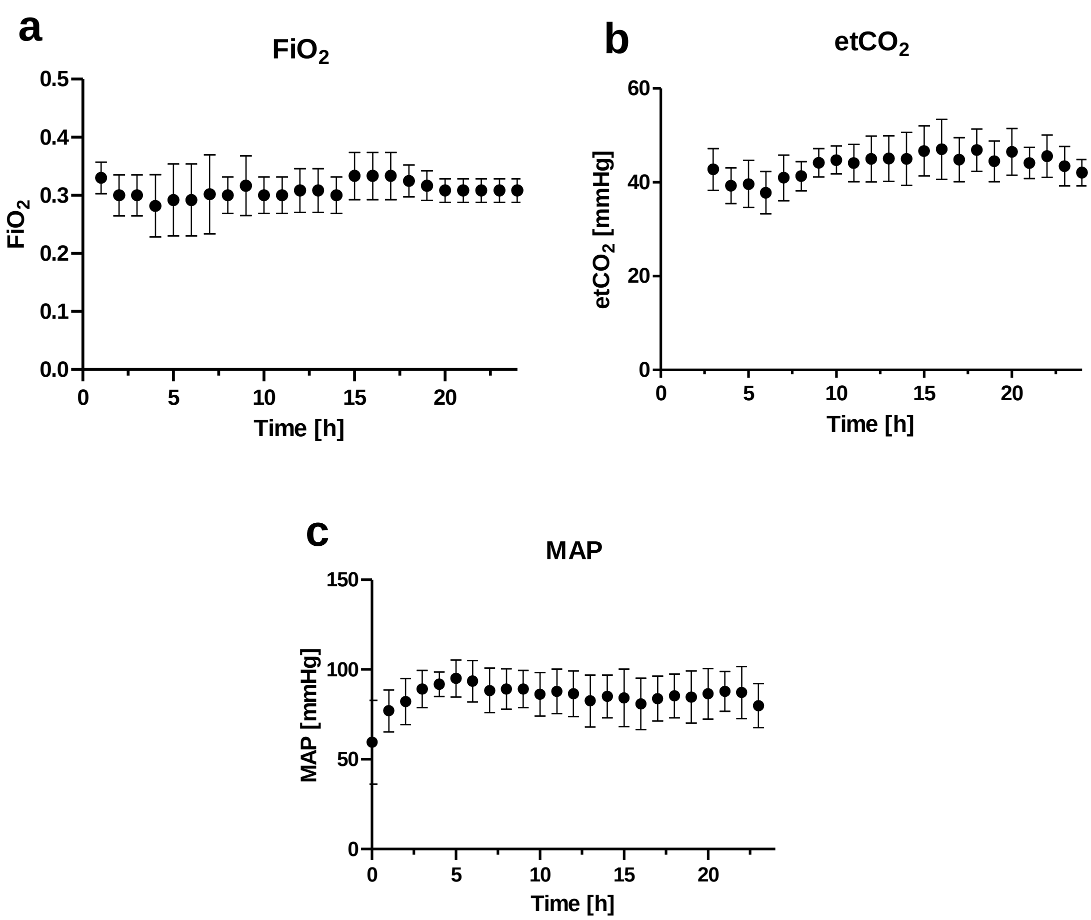

Fig. S3: Inspiratory oxygen fraction (FiO_2), end-expiratory carbon dioxide ($etCO_2$) and mean arterial pressure (MAP)

(a) shows the inspiratory oxygen fraction (FiO_2), (b) the end-expiratory carbon dioxide ($etCO_2$) and (c) depicts the mean arterial pressure (MAP). Data were recorded manually for FiO_2 every 20 minutes and for MAP every 10 minutes throughout the study period. $etCO_2$ was recorded automatically for each breath in the entire stimulated study period. The cumulated mean values with standard deviation per hour were plotted for all six pigs.

Tab. S1 Mean arterial blood gas values

Description	Value
pH	7.46 ± 0.06
pO_2 [mmHg]	144.9 ± 32.8
pCO_2 [mmHg]	42.1 ± 6.6
sO_2 [%]	99.6 ± 1.5
Hb [g/dl]	8.4 ± 0.6
Na^+ [mmol/l]	140.2 ± 2.2
K^+ [mmol/l]	3.9 ± 0.3
Ca^{2+} [mmol/l]	1.35 ± 0.07
Glucose [mmol/l]<br>[mg/dl]	6.2 ± 1.5<br>112 ± 26
Lactate [mmol/l]	0.8 ± 0.7
Base [mmol/l]	4.9 ± 1.6
HCO_3^- [mmol/l]	28.9 ± 1.6

Arterial blood gas analysis was performed every two hours over the stimulated study period. Included are all measurements for all six pigs. Data is shown as mean ± SD.

Tab. S2 Quantitative comparison of intratracheal pressure values within breaths between MV and PNS

Animal No.	Breathing phase	Percentage of mTV	PNS Ptrach [mbar]	MV Ptrach [mbar]	p-value
Pig2	Insp.	25%	2.24 ± 1.68	9.80 ± 0.22	<0.001
		50%	0.69 ± 2.05	11.62 ± 0.24	<0.001
		75%	1.77 ± 1.31	13.53 ± 0.24	<0.001
	Exp.	25%	7.08 ± 0.25	7.74 ± 0.11	<0.001
		50%	9.59 ± 0.16	9.50 ± 0.14	<0.001
		75%	11.36 ± 0.20	11.19 ± 0.18	<0.001
Pig3	Insp.	25%	1.36 ± 0.56	11.25 ± 0.36	<0.001
		50%	2.10 ± 0.34	13.42 ± 0.35	<0.001
		75%	3.79 ± 0.41	15.23 ± 0.26	<0.001
	Exp.	25%	7.43 ± 0.19	7.05 ± 0.31	<0.001
		50%	9.28 ± 0.29	8.53 ± 0.24	<0.001
		75%	10.98 ± 0.22	9.99 ± 0.27	<0.001
Pig4	Insp.	25%	-0.83 ± 0.55	12.36 ± 0.45	<0.001
		50%	0.76 ± 0.68	14.81 ± 0.41	<0.001
		75%	3.15 ± 0.45	16.98 ± 0.34	<0.001
	Exp.	25%	8.01 ± 0.47	7.48 ± 0.44	<0.001
		50%	10.37 ± 0.47	9.26 ± 0.37	<0.001
		75%	12.10 ± 0.40	11.09 ± 0.43	<0.001
Pig5	Insp.	25%	-2.90 ± 0.49	11.95 ± 0.17	<0.001
		50%	-1.65 ± 0.72	14.17 ± 0.09	<0.001
		75%	1.61 ± 1.15	15.88 ± 0.11	<0.001
	Exp.	25%	7.65 ± 0.12	7.19 ± 0.14	<0.001
		50%	9.18 ± 0.22	8.81 ± 0.08	<0.001
		75%	10.90 ± 0.29	10.63 ± 0.12	<0.001
Pig6	Insp.	25%	0.41 ± 0.47	11.71 ± 0.20	<0.001
		50%	1.95 ± 0.42	13.58 ± 0.12	<0.001
		75%	2.37 ± 0.30	14.89 ± 0.05	<0.001
	Exp.	25%	7.09 ± 0.14	6.90 ± 0.10	<0.001
		50%	8.50 ± 0.12	8.22 ± 0.08	<0.001
		75%	10.37 ± 0.30	9.77 ± 0.15	<0.001

Mean intratracheal pressure (Ptrach) values were calculated for 25%, 50% and 75% of the average PNS-induced tidal volume of the respective pig including 100 consecutive breaths for PNS as well as MV separately (n=5). Inspiration (Insp.) and Expiration (Exp.) were analyzed separately. Ptrach is shown as mean ± SD.

Exp.: Expiration, Insp.: Inspiration, mTV: Mean tidal volume, MV: Mechanical ventilation, PNS: Phrenic nerve stimulation, Ptrach: Intratracheal pressure

Video S1: Ultrasound-guided 4-step approach for stimulation needle placement

Bilateral approach of needle placement for phrenic nerve stimulation illustrated by ultrasonic B-mode video-loops with annotations. Displayed is the 4-step approach in a step-by-step manner.

Video S2: Comparison of diaphragmatic contraction for mechanical ventilation and phrenic nerve-stimulated breaths

Bilateral, diaphragmatic ultrasonic B-mode video-loops and external view on the animal thoracic movement with annotations showing a breath-by-breath comparison between mechanical ventilation and phrenic nerve stimulated breaths.

Video S3: Comparison of caudal regional lung ventilation in PNS and MV using EIT

Regional lung ventilation for phrenic nerve stimulated (PNS) breathing and mechanical ventilation (MV) in breath-by-breath alternation using caudally placed electrical impedance tomography (EIT). The video shows the dynamic EIT intensity over time. The stronger the intensity of the EIT-signal, the brighter the blue color. Ventral lung areas are shown at the top, dorsal areas are at the bottom of the respective video.
